# Can we rely on artificial intelligence to guide antimicrobial therapy? A systematic literature review

**DOI:** 10.1017/ash.2025.47

**Published:** 2025-03-31

**Authors:** Sulwan AlGain, Alexandre R. Marra, Takaaki Kobayashi, Pedro S. Marra, Patricia Deffune Celeghini, Mariana Kim Hsieh, Mohammed Abdu Shatari, Samiyah Althagafi, Maria Alayed, Jamila I Ranavaya, Nicole A. Boodhoo, Nicholas O. Meade, Daniel Fu, Mindy Marie Sampson, Guillermo Rodriguez-Nava, Alex N. Zimmet, David Ha, Mohammed Alsuhaibani, Boglarka S. Huddleston, Jorge L. Salinas

**Affiliations:** 1 King Faisal Specialist Hospital and Research Center, Riyadh, Saudi Arabia; 2 Division of Infectious Diseases & Geographic Medicine, Stanford University, Stanford, CA, USA; 3 Hospital Israelita Albert Einstein, São Paulo, SP, Brazil; 4 University of Iowa Hospitals and Clinics, Iowa City, IA, USA; 5 Department of Internal Medicine, University of Kentucky, Lexington, KY, USA; 6 School of Medicine, University of California, San Francisco, San Francisco, CA, USA; 7 King Saud Medical City, Riyadh, Saudi Arabia; 8 Pediatric Infectious Diseases, King Abdullah Specialized Children’s Hospital, MNGHA, Jeddah, Saudi Arabia; 9 Department of Epidemiology, University of Iowa College of Public Health, Iowa City, IA, USA; 10 Pritzker School of Medicine, University of Chicago, Chicago, IL, USA; 11 Lane Medical Library, Stanford University School of Medicine, Palo Alto, CA, USA

## Abstract

**Background:**

Artificial intelligence (AI) has the potential to enhance clinical decision-making, including in infectious diseases. By improving antimicrobial resistance prediction and optimizing antibiotic prescriptions, these technologies may support treatment strategies and address critical gaps in healthcare. This study evaluates the effectiveness of AI in guiding appropriate antibiotic prescriptions for infectious diseases through a systematic literature review.

**Methods:**

We conducted a systematic review of studies evaluating AI (machine learning or large language models) used for guidance on prescribing appropriate antibiotics in infectious disease cases. Searches were performed in PubMed, CINAHL, Embase, Scopus, Web of Science, and Google Scholar for articles published up to October 25, 2024. Inclusion criteria focused on studies assessing the performance of AI in clinical practice, with outcomes related to antimicrobial management and decision-making.

**Results:**

Seventeen studies used machine learning as part of clinical decision support systems (CDSS). They improved prediction of antimicrobial resistance and optimized antimicrobial use. Six studies focused on large language models to guide antimicrobial therapy; they had higher prescribing error rates, patient safety risks, and needed precise prompts to ensure accurate responses.

**Conclusions:**

AI, particularly machine learning integrated into CDSS, holds promise in enhancing clinical decision-making and improving antimicrobial management. However, large language models currently lack the reliability required for complex clinical applications. The indispensable role of infectious disease specialists remains critical for ensuring accurate, personalized, and safe treatment strategies. Rigorous validation and regular updates are essential before the successful integration of AI into clinical practice.

## Background

Artificial intelligence (AI) is defined as the development of computer systems capable of performing actions that usually require human intelligence. AI is rapidly evolving, with applications expanding across numerous fields, including healthcare.^1–3^ Infectious disease (ID) specialists, frequently consulted for guidance on appropriate antimicrobial therapy, provide critical recommendations tailored to specific clinical scenarios. However, there is a national shortage of ID specialists leaving many institutions without any access to their expertise. This results in substantial variability in ID care across healthcare settings with rural communities being particularly negatively impacted. ID specialists are essential to lead nationally mandated antibiotic stewardship programs, yet according to recent data from the CDC, less than 50% of programs have an ID-trained physician leader.^4^

This gap presents an opportunity for AI-based tools, such as large language models (LLMs), and machine learning algorithms, to enhance clinical decision-making.^5^ These technologies are capable of generating rapid responses to clinical queries, offering support in diagnosing conditions and suggesting treatment plans, thereby improving the efficiency of medical practice.^6–8^ Machine learning (ML) holds the potential to revolutionize healthcare by analyzing large datasets to identify patterns and insights that may elude human observation.^3^ A recent study highlighted that, evaluators preferred responses from ChatGPT to those provided by physicians in addressing patient queries, underscoring AI’s ability to deliver high-quality and empathetic responses.^9^ Additionally, ChatGPT has shown promise by accurately answering medicine-related multiple-choice and single-choice questions in standardized assessments.^2^

Despite these advances, concerns remain regarding the variability in performance among AI models, particularly when addressing complex clinical cases such as those that often comprise the bulk of ID physicians’ practice. This highlights the need for rigorous evaluation of AI tools to ascertain their accuracy, reliability, and feasibility for integration into routine medical practice.^9,10^ In this study, we evaluated the performance of ML and LLMs in recommending appropriate antibiotic therapy for various infectious diseases. By comparing these recommendations to those provided by standard care practices, we sought to assess the accuracy, limitations, and clinical applicability of these tools to better understand the potential role and safety of AI in antimicrobial prescribing.

## Methods

### Systematic literature review and inclusion and exclusion criteria

This review was conducted according to the Preferred Reporting Items for Systematic Reviews (PRISMA) statement.^11^ This study was registered on PROSPERO (https://www.crd.york.ac.uk/PROSPERO/) on September 26, 2024(CRD42024594704). Institutional Review Board approval was not required for this work. The review included manuscripts published from the inception of each database to the present, without language restrictions. The literature search covered studies from inception through October 25, 2024. Eligible studies met the following inclusion criteria: original research articles published in peer-reviewed journals, conducted in healthcare settings, and evaluating the use of AI, ML, or clinical decision support systems (CDSS) in managing infectious diseases. ML models, such as random forests and gradient-boosted decision trees, were used to predict antimicrobial resistance and optimize antibiotic selection by analyzing large datasets to uncover actionable patterns. LLMs (eg, ChatGPT) are designed to interpret and generate human-like text. Exclusion criteria included case reports, commentaries, pilot studies, and studies focusing solely on diagnosis without addressing treatment.

### Search strategy

We performed a comprehensive search of the literature in PubMed, CINAHL, Scopus, Web of Science, Embase, and Google Scholar in collaboration with an experienced health sciences librarian (B.H.) (Supplementary Appendix Table 1). Reference lists of retrieved articles were reviewed using Covidence software^12^ to identify additional relevant studies. Two investigators (S.M.A. and A.R.M.) independently screened titles and abstracts, applying the inclusion criteria to exclude irrelevant studies. Discrepancies were resolved by consensus. This systematic review was guided by the PICO framework^13^, focusing on patients with infectious diseases (P), interventions using AI-based management (I), comparisons with standard management provided by usual care providers (C), and primary outcomes (O) including the accuracy, efficacy, and limitations of AI in antimicrobial management.

### Data abstraction and quality assessment

Of the twelve independent reviewers (A.R.M., D.F., J.I.R., M.A., M.K.H., M.A.SH., N.A.B., N.O.M., P.D., P.S.M., S.TH., T.K.), two independently abstracted data from each included study using a standardized data collection form (supplementary appendix). Extracted data included study design, publication year, calendar period, AI methodology, and comparisons with usual care providers where applicable. Reviewers also documented sensitivity and specificity of AI models, clinical impact, advantages, and limitations.

Risk of bias was assessed using a modified version of the Downs and Black scale.^14^ The scale, with a maximum possible score of 28, evaluates quality across domains including reporting, internal validity, and external validity. The reviewers independently scored each study, resolving discrepancies through consensus.

## Results

### Characteristics of included studies

**Figure 1.**
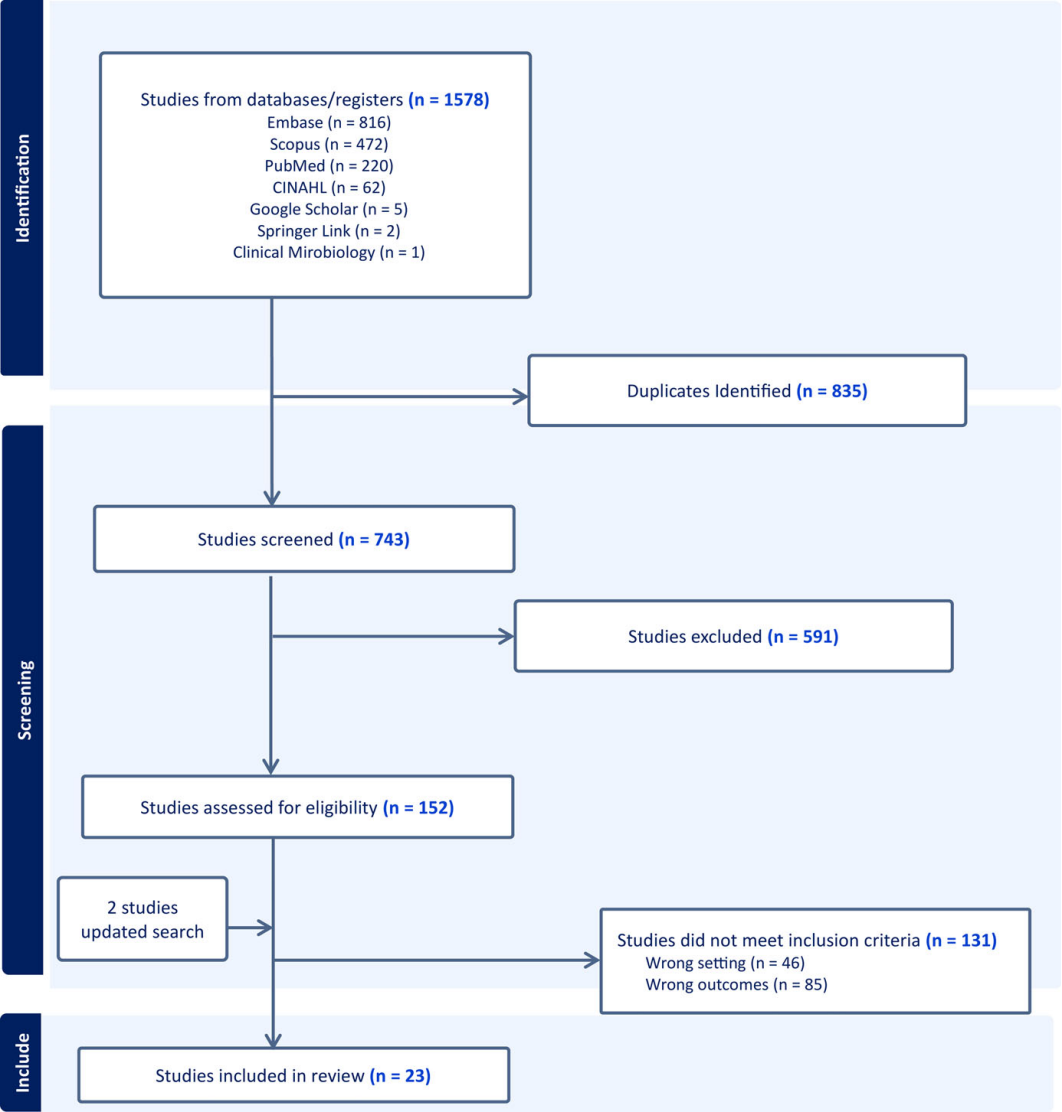
Literature search for articles that evaluated the performance and effectiveness of artificial intelligence or machine learning in recommending appropriate antibiotics for various infectious diseases.

1,578 articles were retrieved. After applying exclusion criteria, 154 studies were reviewed in full, of which 23 met the inclusion criteria^15–37^ and were included in the final analysis (Figure 1). These included eleven cohort studies (nine retrospective and two prospective), three qualitative studies, two cross-sectional studies, two quasi-experimental studies, and five randomized control studies (Table 1). Of these, 17 studies focused on AI applied as ML algorithms integrated into clinical decision support systems (CDSS) to enhance clinical outcomes^15–28,36,37^, while the remaining six studies evaluated various LLMs^29–34^, including ChatGPT^29–33^. Geographically, six studies were conducted in the United States^21,23,24,28,36,37^, three in South Korea^15,18,22^, and one each in Austria^29^, Australia^35^, Cambodia^20^, Canada^21^, China^32^, France^33^, Germany^19^, Israel^16^, Italy^30^, Tanzania^27^, Turkey^31^, the Netherlands^26^, Switzerland^34^, the United Kingdom^25^, and Vietnam.^17^ The studies were conducted between 2017 and 2024, with durations ranging from two weeks to ten years. The studies examined AI in two domains (Table 2). The first included seventeen studies exploring the integration of AI into CDSS, focusing on antimicrobial resistance prediction, the appropriateness of antibiotic prescriptions, antimicrobial stewardship, and the transition from intravenous to oral antibiotic therapy. The second domain involved six studies that evaluated the performance of LLMs in addressing infectious disease management, highlighting both successes and limitations across a range of conditions (Table 2).

**Table 1.** Characteristics of included studies (N = 23)

Category	Description
**Study Types**	- Cohort studies: 11 (9 retrospective, 2 prospective); - Qualitative study: 3; - Cross-sectional studies: 2; - Quasi-experimental studies: 2; - RCT: 5
**Focus on AI Applications**	- Active Machine Learning (ML) in Clinical Decision Support Systems (CDSS): 17 studies - Large Language Models (LLMs), including ChatGPT: 6 studies
**Geographic Locations of Studies**	- USA: 6 studies^21,23,24,28,36,37^ - South Korea: 3 studies^15,18,22^ - Austria^29^, Australia^35^, Cambodia^20^, Canada^21^, China^32^, France^33^, Germany^19^, Israel^16^, Italy^30^, Tanzania^27^, Turkey^31^, the Netherlands^26^, Switzerland^34^, United Kingdom^25^ and Vietnam^17^: 1 study each
**Study Duration**	Ranged from 2 wk to 10 yr (2017–2024)
**AI in Clinical Decision Support Systems (CDSS)**	**Antimicrobial Stewardship** 5 studies utilizing models like XGBoost, LightGBM, eXtreme and computerized provider order entry (CPOE) for ASP improvements^22–24,36,37^ **Antimicrobial Resistance Prediction** 4 studies evaluating AI algorithms for antibiotic resistance prediction^15–18^ **Antibiotic Prescription Appropriateness** 3 studies evaluating CDSS for improving antibiotic prescribing accuracy^19–21^ **Accuracy of Infection Management** 2 studies on improved UTI management through CDSS and RF models^26,27^ 1 study on adequate and optimal management of OM in children through Bayesian Network^35^ **IV to Oral Antibiotic Transition** 2 studies on early switch protocols^24,25^ **Adherence to Clinical Guidelines** 1 study promoting guideline adherence in pediatric pneumonia^28^
**Large Language Models (LLMs)/without CDSS**	6 studies examining LLM performance in infectious disease management for prosthetic joint infections, endocarditis, abdominal infections, bloodstream infections, UTIs, and meningitis^29–34^
**Study Quality Assessment**	- High quality (Downs & Black score 19–22): 15 studies^17,18,20–22,25–30,33,35–37^ - Fair quality (score 14–18): 6 studies^16,19,23,24,32,34^ - Low quality (score ≤13): 2 studies.^15,31^

**Table 2.** Summary of characteristics of studies included in the systematic review

First author, year, location	Study type	Duration	Topic	AI application	Focus area	Model used	Key findings	Limitations and risks	Quality score
CDSS									
Bolton^25^ 2024, London UK	Retrospective Cohort	2 yr	Adult, UTI, Sepsis, Pneumonia	ML in CDSS	Early switch from IV to oral antibiotics	Short & long models using MIMIC/eICU (medical information mart for intensive care	Models provided individualized prediction for when a patient can change from IV to oral	Not all patient’s factors were clinically used to assess sustainability of switching to oral	20
Ciarkowski^24^ 2020, Utah, USA	Quasi experimental	18 mo	Adult, CAP	ML in CDSS for ASP advise	Impact of CDSS tool, when linked with a clinical pathway for CAP	CDSS in three phases (education, with active ASP, advisory only)	Length and cost of antibiotics were reduced with active ASP	Duration with active ASP was limited	17
Ghamrawi^23^ 2017, Ohio, USA	Quasi experimental	3 mo	ASP	ML in CDSS	To generate ASP alerts for ID clinical pharmacists	CDSS (TheraDoc)	Time to change antibiotics was better in postimplementation phase	Alert fatigue	18
Gohil^36^, 2024, USA	RCT	36 mo	ASP, CAP	ML in CDSS for ASP	To generate prompts that predicts MDRO risk	CPOE	Empiric antibiotic days were significantly lower in the CPOE bundle	Cultures were included without considering specimen quality. Concurrent prompts for UTI and pneumonia may have caused alert fatigue, potentially impacting both systems.	23
Gohil^37^, 2024, USA	RCT	35 mo	ASP, UTI	ML in CDSS for ASP	To generate prompts that predicts MDRO risk	CPOE	Empiric antibiotic days were significantly lower in the CPOE bundle	All positive cultures were included regardless of colony count, and assigning risks below 10% was deemed overly conservative	23
Herter^26^ 2022, Amsterdam, Netherlands	Prospective Cohort	4 mo	Adult UTI	ML in CDSS	Compare the proportion of successful treatment before and after implementation of CDSS	Pacmed database	5% increase in successful treatment rate	Therapy compliance cannot be mitigated through this system.	19
Ilhani^15^ 2024, Yongin, South Korea	Retrospective Cohort	120 mo	Adult UTI	ML in CDSS	Evaluate ML algorithms for predicting antimicrobial resistance in patients with UTI	CDSS: random forest	Aimed to assist clinicians in selecting the correct antibiotics	There was a lack of MDR dataset when building the model	11
Lee^18^ 2023, Incheon, South Korea	Retrospective Cohort	18 mo	Adult UTI	ML in CDSS	Predicting Ciprofloxacin and ESBL resistance	GBDT	Sensitivity to predict Ciprofloxacin resistance and ESBL, were 93% and 94% respectively	Rapidly evolving resistance can make this system outdated	24
Lewin-Epstein^16^ 2021, Tel Aviv, Israel	Retrospective Cohort	32 mo	Adult AMR	ML in CDSS	Predict antimicrobial resistance	L1 regularized logistic regression, GBDT, and neural network models	The ensemble outperformed the separate models and produced accurate predictions	There was a concern as no mention to comparison to physician predictions.	16
MacFadden^21^ 2018, Toronto, Canada and Chicago, USA	Retrospective Cohort	60 mo	Adult Gram-negative BSI	ML in CDSS	Help narrow antibiotics spectrum	Model AUC	Aided in appropriate antibiotic selection with incorporated specific patients’ characteristics and prior to culture results	The authors did not use a split- sample model	22
Neugebauer^19^2020, Mannheim, Germany	RCT	2 yr	Adult upper UTI	ML in CDSS	System was integrated with national guidelines	Antibiotix	Provided correct diagnosis, and suggested proper therapy	Its integration to clinical workflow may be challenging	14
Oonsivilai^20^ 2018, Siem Reap, Cambodia	Retrospective Cohort	36 mo	Pediatric antimicrobial resistance	ML in CDSS	Predict AMR	Random forest	The model performed well for ceftriaxone	The model may be less relevant as time passes.	21
Quoac^17^ 2023, Ho Chi Minh City, Vietnam	Cross-sectional	29 mo	Adult AMR	ML in CDSS	Predict AMR	Random forest, XBoost, LightGBM	The models made accurate predictions	The results need the coordination with clinicians for proper accuracy	20
Tan^27^ 2023, Mbeya, Tanzania	RCT	11 mo	Antibiotics prescription in pediatric	ML in CDSS	Evaluate the impact of CDSS in antibiotics prescription and day 7 clinical outcome	ePOCT+	Decreased total antibiotic prescriptions	7 d is the usually the natural course of diseases, which may affect outcome of study,	25
Tran-The^22^ 2024, Seoul, South Korea	Retrospective Cohort	14 mo	ASP	ML in CDSS	Antibiotics discontinuation, switch to oral and de-escalation of antibioitcs	XGB and LGBM	Significant early discontinuation and switch from IV to oral	Some cases had low severity infection index, which may affect the results.	19
Williams^28^ 2023, Tennessee, USA	RCT	4 wk	Pediatric Pneumonia	ML in CDSS	Guideline concordance for antibiotic prescribing for pneumonia	HER-based antibiotic advisor	Earlier discharge and switch to oral antibiotics	Alert fatigue.	20
Wu^35^ 2020. Perth, Australia	Retrospective Cohort	Not reported	Pediatrics osteomyelitis	ML in CDSS	Accurate pathogen detection and improving antibiotics selection	Bayesian network	Recommended antibiotics were rated optimal or adequate by experts	The model with data only, did not provide accurate pathogen prevalence.	19
ChatGPt and Large language models									
Cakir^31^ 2024, Istanbul, Turkey	Cross sectional	2 wk	Adult UTI	ChatGPT	Accuracy and proficiency of ChatGPT	Questions for UTI guidelines and management	High accuracy with more than 90% compliance to national guidelines	The remaining incorrect answers may have serious public health outcomes	13
De Vito^30^2024, Italy	Qualitative	Not reported	Endocarditis, Pneumonia, Intra-abdominal infections, and BSI)	ChatGPT4	Evaluate accuracy and completeness of responses	6 true or false questions, 6 open ended questions, 7 clinical cases with antibiograms	Excelled in generating responses to structured queries, but performance in complex scenarios was limited.	Difficulty of the questions were subjective to the experts answering or provided the questions.	22
Draschl^29^, 2023, Graz, Austria	Retrospective Cohort	Not reported	Management of PIJ of hip and joint	ChatGPT	Accuracy of answers	There were 27 questions in total, in which 10 were related to treatment via antibiotics	Thorough response related to duration of antibiotics	It prescribed less reliable antibiotics.	19
Fisch^34^, 2023 Basel, Switzerland	Qualitative	Not reported	Adult meningitis	7 different large language models	Adherence to good clinical practice and two international guidelines	Bard, Bing, Claude-2, GTP-3.5, GTP-4, Llama, PaLM)	They were able to determine early initiating of antibiotics, but with low accuracy and appropriateness for coverage	It needs a step by stem reasoning.	17
Maillard^33^ 2023, Paris, France	Prospective Cohort	4 wk	Adult BSI	ChatGPT	If ChatGPT can replace ID consultants	GPT-4	Optimal and appropriate in only one case with offering optimal but may be inappropriate suggestions in 17 cases	Sensitive to prompt	23
Tao^32^, 2024, Guangzhou, China	Qualitative	Not reported	Infections in vulnerable population	ChatGPT	Accuracy of antibiotics prescription in its type, dose and length	GPT-4	Accurate and comprehensive responses were found in less than 39% of the questions	High error rate, especially in children	14

AI, artificial intelligence; AMR, antimicrobial resistance; ASP, antimicrobial stewardship; AUC, area under the curve; BSI, bloodstream infection; CAP, community-acquired pneumonia; CDSS, clinical decision support systems; CPOE, computerized provider order entry; GBDT, Gradient Boosted Decision Tree; GBDT, Gradient Boosted Decision Tree; HER, health electronic records; IV, intravenous; LLM, large language model; ML, machine learning; PJI, periprosthetic joint infection; RCT, randomized clinical trial; UTI, urinary tract infection.

#### AI in clinical decision support systems: antimicrobial stewardship (ASP)

Five studies demonstrated substantial potential of AI in enhancing antimicrobial stewardship.^22–24,36,37^ One study found that ML models identified 60% of cases for antibiotic discontinuation, compared to 19% in usual care, with a 98% success rate in transitioning to oral antibiotics.^22^ Another study highlighted that AI systems shortened antibiotic de-escalation by 24 hours.^23^ A third study showed that CDSS combined with active ASP achieved better antibiotic optimization for community-acquired pneumonia (CAP) compared to the absence of ASP.^24^ Additionally, two INSPIRE (Intelligent Stewardship Prompts to Improve Real-Time Empiric Antibiotics Section) randomized clinical trials evaluated the impact of a CPOE (computerized provider order entry) bundle versus routine ASP on empiric antibiotic prescribing for pneumonia and urinary tract infection (UTI) in non-critically ill adults.^36,37^ The CPOE reduced extended-spectrum antibiotic therapy days by 28.4% for pneumonia and 17.4% for UTIs, with similar reductions for vancomycin and antipseudomonal use. No significant differences in safety outcomes or ICU transfers were observed in either trial.

### Antimicrobial resistance prediction

Four studies evaluated AI’s use in predicting antimicrobial resistance.^15–18^ One study focused on random forest ML models to predict antibiotic resistance in UTIs, achieving area under the receiver operating characteristic curves (AUROCs) ranging from 0.777 for cephalosporin to 0.884 for fluoroquinolones, with fluoroquinolones showing superior performance.^15^ Another study employed a CDSS integrated with ML and electronic health records, achieving AUROCs of 0.8 to 0.88 for resistance prediction when bacterial species data were included.^16^ In the intensive care, a third study found that a random forest model had high specificity (0.84–0.99), while XGBoost and LightGBM demonstrated better sensitivity (up to 0.95).^17^ A final study used gradient-boosted decision trees to predict ciprofloxacin resistance and extended-spectrum beta-lactamase (ESBL) production in patients with UTI, achieving high sensitivity (∼93%) but lower specificity (31–45%).^18^ These findings show that machine learning performance varies with clinical context, objectives, and hospital resistance patterns.

### Appropriateness of antibiotic prescriptions

Three studies evaluated AI-enhanced CDSS systems designed to improve antibiotic prescribing.^19–21^ One study demonstrated that a CDSS integrated with national guidelines improved diagnostic accuracy and reduced unnecessary antibiotic use in UTIs, increasing prescriber confidence.^19^ A second study employed a random forest model within a CDSS to guide antibiotic selection for pediatric infections, particularly improving predictions of ceftriaxone resistance, with an estimated AUROC of 0.80.^20^ A third study assessed a CDSS leveraging patient data and prior cultures to improve empiric therapy for gram-negative bloodstream infections, enabling narrower-spectrum antibiotic use in 78% of cases while reducing inadequate treatment. Its impact varied with antibiotic susceptibility thresholds, and multivariable models showed good discrimination, with AUROCs of 0.68–0.89 for Gram-stain-guided and 0.75–0.98 for pathogen-guided models.^21^

### Accuracy of management

Three studies examined the accuracy of AI-enhanced CDSS in managing infections.^26,27,35^ One study reported a 5% improvement in treatment success for UTIs (defined as the absence of antibiotic prescriptions within 28 d) compared to usual care.^26^ Another study demonstrated that integrating CDSS with point-of-care testing significantly reduced antibiotic prescriptions by (∼47%) on day 0, contributing to improved antimicrobial stewardship.^27^ A third study used a Bayesian network to guide personalized therapy for osteomyelitis in children, achieving expert-rated optimal or adequate recommendations in 82%–98% of cases, despite initial underestimation of *Staphylococcus aureus* prevalence.^35^

### Transition from intravenous to oral antibiotics

Two studies focused on AI-assisted transitions from intravenous to oral antibiotics.^24,25^ One study, conducted in three phases, demonstrated that a CDSS facilitated earlier conversion to oral antibiotics, reducing the duration of intravenous therapy and achieving a 20% cost reduction without affecting length of stay.^24^ Another study used machine learning to individualize the transition, reducing the Antimicrobial Spectrum Index by a mean of 23%, though variability in outcomes was noted.^25^

### Adherence to guidelines

One study evaluated AI-based CDSS for promoting guideline-concordant antimicrobial prescriptions in pediatric patients with community-acquired pneumonia. The system improved adherence to guidelines by 10% compared to usual care and facilitated faster initiation of antibiotics.^28^

#### Large language models:

Six studies examined the use of AI across various LLMs.^29–34^ While AI language models performed well on straightforward queries, they struggled with complex cases that required nuanced clinical judgment. Nevertheless, three studies found consistent results for simple queries, with responses receiving high scores on different scales such as Likert and Global Quality Scales.^29–31^

### Accuracy of management:

Five studies assessed the accuracy of various LLMs in responses related to management.^29,30,32–34^ One study found that AI performed poorly on management-related questions for prosthetic joint infections, achieving less than 45% accuracy in such scenarios. While significant inter-rater reliability was observed for responses on diagnosis and treatment, these responses received the lowest scores in this subtopic of treatment, indicating low trustworthiness.^29^ In another study examining various clinical scenarios, including CAP, bloodstream infections, endocarditis, and meningitis, GPT-4.0 demonstrated about 70% accuracy in true/false questions. However, its performance greatly declined on more complex questions, with accuracy dropping to approximately 37.5%. Subtopic analysis showed over 80% accuracy in endocarditis cases but less than 50% in CAP cases. Additionally, the AI often recommended overtreatment and failed to consider newer antibiotics, achieving only 10% accuracy in complex cases like endocarditis and bacteremia.^30^ AI also faced challenges with infections in vulnerable populations, delivering incomplete or incorrect answers in up to 80% of pediatric cases. Error rates for treatment responses across children, pregnant individuals, adults, those with drug allergies, and patients with chronic kidney disease ranged from 11% to 44%, with the highest inaccuracies observed in pediatric-related questions.^32^ In a study evaluating ChatGPT’s responses for managing bloodstream infections, it provided appropriate and optimal suggestions in about 35% of cases. However, it offered inadequate or potentially harmful recommendations in 3% to 34% of cases for both definitive and broad-spectrum therapies. The remaining responses were deemed appropriate but not optimal.^33^ In one study evaluating the accuracy of seven different LLMs in managing meningitis, GPT-4 showed the most consistent performance, providing over 80% correct answers across all tasks (beyond just treatment suggestions). When asked about the correct empirical treatment, the models provided accurate suggestions in approximately 38% of cases, with Claude-2 and GPT-4 performing the best. Specifically, in recommending the addition of antivirals, only 33% of the models suggested it, with just half providing the correct dosage. Over 60% of the models opted not to provide a dosing recommendation.^34^

### Adherence to guidelines

Two studies assessed AI’s adherence to guideline.^31,34^ For UTI, LLMs demonstrated 87% adherence to guidelines but produced incorrect responses in >10% of cases, raising safety concerns.^31^ One study noted that adherence to guidelines across seven LLMs ranged from 53% to 85%, with lower task completion correlating to reduced response consistency.^34^

### Antimicrobial resistance:

One study revealed that untrained GPT-4.0 demonstrated lower accuracy in identifying the correct resistance mechanism, with the best-suggested answers being in the subtopic of community-acquired pneumonia.^30^

#### Quality assessment

Using the Downs and Black tool, 15 studies were rated as high quality, with scores ranging from 19 to 25 out of 28 points.^17,18,20–22,25–30,33,35–37^ Six studies were rated as fair, scoring between 14 to 18 points^16,19,23,24,32,34^, and two were classified as low quality, scoring 13 or fewer points.^15,31^ Detailed results are available in the Supplementary Material.

## Discussion

In this systematic review, we evaluated the effectiveness of artificial intelligence (machine learning and large language models) in guiding appropriate antibiotic prescriptions for various infectious diseases. This review showed that AI, particularly when integrated into clinical decision support, can enhance clinical decision-making by improving antimicrobial resistance predictions and optimizing antibiotic selection. Seventeen studies illustrated the positive impact of AI in reducing unnecessary antibiotic use and improving treatment outcomes. However, LLMs, such as ChatGPT, performed markedly less effectively in complex management scenarios, frequently producing substantial errors that could compromise patient safety. These contrasting results emphasize the importance of context when implementing AI tools in clinical practice, reinforcing the critical need for consultation with infectious disease specialists to ensure accurate and individualized treatment strategies.

Previous studies explored the performance of ChatGPT in responding to multiple-choice and single-choice questions, demonstrating superior accuracy in single-choice formats^2^. However, as case complexity increased, accuracy declined significantly, a pattern consistent with our findings. While AI, including ChatGPT, holds promise in certain applications, its use in clinical settings must be approached with caution. Success in one domain does not necessarily translate across all specialties. Although AI-powered chatbots can deliver detailed drug information, many responses have been found to be inaccurate or potentially harmful, as documented in previous research.^4,6^ The main challenges to implementing LLMs in clinical practice are their lack of situational awareness, inference ability, and consistency, which could jeopardize patient safety.^39^ This aligns with the results of our review.

The studies included in this analysis assessed AI’s role in optimizing antibiotic prescriptions^19–21^, adherence to guidelines^28^, accuracy in infection management^26,27,35^, antimicrobial stewardship^22–24,36,37^, and facilitating early transitions from intravenous to oral antibiotics.^23–25^ A total of 23 studies were analyzed, including 18 non-randomized and five randomized studies. The AI systems varied considerably in their methodologies, with half of the studies demonstrating moderate methodological quality, while the remainder were rated fair to low. Our findings suggest that ML algorithms perform well in predicting antimicrobial resistance, with high sensitivity rates. This capability allows for earlier narrowing of antibiotic choices before culture results are available. However, the low specificity of these models necessitates cautious interpretation.^15–18^

AI also showed promise in facilitating early transitions from intravenous to oral antibiotics, potentially reducing hospital stays, and possibly lowering healthcare costs.^24,25^ However, these systems face significant limitations. For instance, high error rates were observed in complex clinical cases (especially evident in LLM) and while AI occasionally flagged contraindicated antibiotics, this function was inconsistently applied. Incorrect antibiotic recommendations in such cases could result in serious, even fatal, outcomes. Furthermore, the integration of AI into hospital systems requires substantial resources, including manpower, financial investment, and technical infrastructure, which may not be feasible in all healthcare settings. Excessive alerts generated by AI systems can lead to alert fatigue, a phenomenon well documented in prior studies^23,36–38^ potentially diminishing trust and engagement among healthcare providers. While promising, LLM agents present risks and safety concerns, bias, over-reliance, and the need for strong regulation.^39^ Liability guidelines must evolve to address the dynamic nature of LLM-based systems, which adapt and evolve through interactions with external resources. Current static regulations fall short, requiring proactive measures to anticipate issues and failures.^39^

This review has several limitations. First, the majority of the included studies were non-randomized, and non-blinded which could affect the reliability and robustness of the findings. Second, there was a lack of focus on pediatric populations; only three studies specifically addressed pediatric groups, while one included vulnerable population, including children, but was not exclusively focused on them, limiting the generalizability of the findings to this demographic. Third, most studies did not directly compare AI-driven management strategies with recommendations from ID specialists, thus their role in the context of existing medical resources may remain inconclusive, which may have influenced the accuracy assessments. Fourth, while four studies evaluated antimicrobial resistance, the rapidly evolving nature of resistance poses challenges to the sustained relevance of AI models, potentially limiting their future applicability, and limiting their long-term utility. Furthermore, variability in expert opinion complicates the interpretation of AI-generated recommendations. Additionally, most included studies lacked detailed descriptions of the source materials used for AI tool development and inconsistently reported the incorporation of patient-specific parameters, limiting the evaluation of potential biases and the extent to which these tools fulfill the promise of personalized medicine. Finally, while clinical decision support can optimize antibiotic dosing by incorporating electronic health record data, such as renal and liver function, the high costs associated with implementing these systems may limit their accessibility, particularly in resource-constrained settings. Additionally, while we used the Downs and Black checklist for quality assessment^14^, which is validated for clinical studies, the Roosan D. checklist^40^, has not been validated for studies involving LLMs, which are included in our analysis.

In conclusion, AI holds promise, particularly in predicting antimicrobial resistance and optimizing antibiotic use. However, its current limitations highlight the essential role of infectious disease specialists in providing precise, personalized, and comprehensive care. The safe and effective integration of AI into clinical practice will depend on rigorous validation, continuous updates, and close collaboration with human expertise to ensure optimal outcomes.

## Supporting information

AlGain et al. supplementary material AlGain et al. supplementary material

## Supplementary material

For supplementary material accompanying this paper visit http://doi.org/10.1017/ash.2025.47. click here to view supplementary material
