# Supplementary material for: Can we rely on artificial intelligence to guide antimicrobial therapy? A systematic literature review
Source: Antimicrob Steward Healthc Epidemiol. 2025 Mar 31;5(1):e90. doi: 10.1017/ash.2025.47 (PMC11986881; doi:10.1017/ash.2025.47)
Supplement: AlGain et al. supplementary material [file S2732494X25000476sup001.docx]

**Can We Rely on Artificial Intelligence to Guide Antimicrobial Therapy?
A systematic literature review**

Sulwan Mujahid AlGain, MD; Alexandre R. Marra, MD; Takaaki Kobayashi, MD; Pedro S. Marra, BS; Patricia Deffune Celeghini, MD; Mariana Kim Hsieh, MD; Mohammed Abdu Shatari, MD; Samiyah Althagafi, MD; Maria Alayed, MD; Jamila I Ranavaya, MD; Nicole A. Boodhoo, BS; Nicholas O. Meade, D.O; Daniel Fu, BS; Mindy Marie Sampson, DO; Guillermo Rodriguez-Nava, MD; Alex N. Zimmet, MD; David Ha, PharmD; Mohammed Alsuhaibani, MD; Boglarka S.Huddleston, MA, MLIS; Jorge L. Salinas, MD.

Running title: Reliability of AI in antibiotic prescriptions

Keywords: machine learning; artificial intelligence; antibiotic prescription; infectious diseases; clinical decision support SYSTEMS

Contents

1. Cover Page
2. Supplemental Appendix Table 1. Search terms and strategy… P.2
3. Mesh Search, PubMed… P.3
4. Supplemental Form 1 : Standardized Data Abstraction Form P.5-22

**Supplementary Appendix Table 1. Search terms and strategies PubMed**

| SEARCH STRING | RESULTS |
| --- | --- |
| S1 – "AI"[Text Word] OR "artificial intelligence"[Text Word] OR "chatbot"[Text Word] OR "chat gpt"[Text Word] OR "machine learning"[Text Word] OR "clinical decision support"[Text Word] OR "decision support system"[Text Word] | 237,688 |
| S2 – "Artificial Intelligence"[Mesh] | 200,341 |
| S3 – "antibiotic* prescription*"[Text Word] OR "antibiotic* therapy"[Text Word] OR "antibiotic prescription"[Title/Abstract:~2] OR "antibiotic therapy"[Title/Abstract:~2] OR "infection management"[Title/Abstract:~2] | 54,977 |
| S4 – "Anti-Bacterial Agents/administration and dosage"[Mesh] OR "Anti-Bacterial Agents/therapeutic use"[Mesh] OR "Antimicrobial Stewardship"[Mesh] | 236,743 |
| S5 – "infectious disease"[Text Word] OR “bacterial infection*”[Text Word] | 89,486 |
| S6 – "Bacterial Infections/diagnosis"[Mesh] OR "Bacterial Infections/drug therapy"[Mesh] OR "Communicable Diseases/drug therapy"[Mesh] OR "Communicable Diseases/therapy"[Mesh] | 579,603 |
| S7 – (S1 OR S2) AND (S3 OR S4) AND (S5 OR S6) | 109 |

**Mesh search PubMed**

((("AI"[Text Word] OR "artificial intelligence"[Text Word] OR "chatbot"[Text Word] OR "chat gpt"[Text Word] OR "machine learning"[Text Word]

OR "clinical decision support"[Text Word] OR "decision support system"[Text Word]) AND (("antibiotic* prescription*"[Text Word] OR "antibiotic*

therapy"[Text Word] OR "antibiotic prescription"[Title/Abstract:~2] OR "antibiotic therapy"[Title/Abstract:~2] OR "infection

management"[Title/Abstract:~2]) AND ("infectious disease"[Text Word])) OR ((("Artificial Intelligence"[Mesh]) AND ((( "Anti-Bacterial

Agents/administration and dosage"[Mesh] OR "Anti-Bacterial Agents/therapeutic use"[Mesh] )) OR ("Antimicrobial Stewardship"[Mesh]))) AND

((( "Bacterial Infections/diagnosis"[Mesh] OR "Bacterial Infections/drug therapy"[Mesh] )) OR (( "Communicable Diseases/drug therapy"[Mesh]

OR "Communicable Diseases/therapy"[Mesh] ))))

(("AI"[Text Word] OR "artificial intelligence"[Text Word] OR "chatbot"[Text Word] OR "chat gpt"[Text Word] OR "machine learning"[Text Word] OR

"clinical decision support"[Text Word] OR "decision support system"[Text Word]) AND (("antibiotic* prescription*"[Text Word] OR "antibiotic*

therapy"[Text Word] OR "antibiotic prescription"[Title/Abstract:~2] OR "antibiotic therapy"[Title/Abstract:~2] OR "infection

management"[Title/Abstract:~2]) AND ("infectious disease"[Text Word])

(("AI"[Text Word] OR "artificial intelligence"[Text Word] OR "chatbot"[Text Word] OR "chat gpt"[Text Word] OR "machine learning"[Text Word]

OR "clinical decision support"[Text Word] OR "decision support system"[Text Word]) AND (("antibiotic* prescription*"[Text Word]) OR

("antibiotic prescription"[Title/Abstract:~2] OR "infection management"[Title/Abstract:~2]))) AND ("infectious disease"[Text Word])

**Supplementary Form1. Data Abstraction Form**

Can We Rely on AI/ML in Replacing Infectious Diseases Physicians for Antibiotic Prescriptions? A Systematic Review

1. First author last name: _________________________________________________
2. Name of article: ________________________________
3. Publication year: ______________________________________________________
4. Study location (City, State, Country):___________________________
5. Reviewer’s initials: _____________________________________________________
6. Study record (leave this to be filled by Sulwan Algain) ___________________

***Please note: some questions can be labeled as N/A for a certain study, just kindly write down N/A.***

Part 1.

Basic Inclusion/Exclusion Criteria:

1. Did the study evaluate AI/ML (machine learning) AND antibiotics? □ Yes □No (if no, exclude it)
2. Did the study evaluate AI/ML vs. Infectious Diseases Physicians? □ Yes □No (if no, we will not exclude it. We will keep this information to make a decision in the future. It will depend on the number of studies that we have found)
3. Duration of study:__________(in weeks or months)
4. Do you believe this study should be excluded? □ Yes □No

If yes, why? _______________________________________________________________

Part 2.

Exposure and Outcomes Assessment (Check more than one if it is necessary)

1. Where did the study take place?

□ An academic medical center

□ A community hospital

□ A nursing homes

□ Other: ______________________________________

1. Which type is this study design?

□ Retrospective Cohort study

□ Prospective Cohort study

□ Case-control study

□ Quasi-experimental study

□Randomized Controlled Trial

1. Was the study performed in more than one hospital? If yes, please add the number of hospitals

- Yes (If yes, please add the # of hospitals) ___ □ No

4. What types of clinical decision support systems (CDSS) or models are evaluated in the included studies for improving healthcare decision-making?”

- ChatGpt
- CDSS, Random Forest Model with or without AUROC (area under the receiver operating characteristic curve)
- Electronic health records-based CDSS
- CDSS. Served based softwares integrated with national Guidelines (different modules are accepted in this option. *Ex: CDSS + national guidelines, CDSS with a standardized SET, to develop a new disease pathway with multidisciplinary team including physicians, pharmacists and engineers)*
- Other (an AI/ML that you feel does not fit into mentioned categories) …………

5. Population characteristics of patients with infectious diseases (Fill out only IF the information is available on the papers)

|  | **AI/ML+/- prescriber** | **Provider (includes physicians/NP/trainee/ASP)** |
| --- | --- | --- |
| Total number and % if mentioned |  |  |
| Age (Mean [SD] or Median [IQR]):  Include number of pediatric patients if any. |  |  |
| Socioeconomic status (if mentioned) |  |  |

6.Parameters and entries of patients with infectious diseases (fill out only IF the information is available)

|  | **AI/ML+/- prescriber** | **Provider (includes physicians/NP/trainee/ASP)** |
| --- | --- | --- |
| Type pf infection (percentage or mean/median of mentioned type)   - Meningitis - UTI - Blood infection - Respiratory infection |  |  |
| Most common Organism (please write down its name with % if mentioned) |  |  |
| Name of top 3 antibiotics studied in % if mentioned (Kindly mention the name and percentage) |  |  |
| Length of antibiotics (mean /median) if mentioned |  |  |
| Early switch to oral (% and days if mentioned) |  |  |
| Length of stay (median/mean in days) If mentioned |  |  |
| Inpatient/outpatient/ ER setting(yes/no) if mentioned |  |  |
| Cost (median/ mean) if mentioned |  |  |

7. How did AI compare to Physicians prescription? (*Which is better, AI+ prescribers or physicians alone, and state reason if mentioned in the article)*

______________________________________________________________________________________________________________________________________________________

8. Were there advantages of AI/ML compared to physicians’ antibiotics prescription for patients: □ Yes □No (*Describe the conclusions of this advantage or not)*

_________________________________________________________________________________________________________________________________________________

9. What kind of Limitations/barriers or challenges did the authors mention when using AI/ML?__

____________________________________________________________________________________________________________________________________________________________________________________________________________________________________

10. Did the study review the reasoning behind AI/ML use? (Why did they use or try to study ML )(some studies were to predict resistance patterns, others to compare accuracy between prescriber and/or machine learning programs, some were to document earlier IV to oral antibiotics, etc…). I*f the answer is YES, please mention the reasoning.*

□Yes □No

________________________________________________________________________________________________________________________________________________________________________________________________________________________________________________________________________________________________________________

11. Was the diagnostic/therapeutic accuracies described for either AI/ML in comparison to human physicians? ( Please note that some articles would not do comparison between a physician and/or machine learning, and the study would focus on assessing advantages of a machine learning device only. If so, please answer question as yes and state that fact, that it was only concerning machine learning with no comparison to a prescriber).If the answer is YES, please mention the reasoning.- *Examples*: *discussing cofounding factors, talking about study design and its limitation, analysis of patient variability, variability of parameters studied, study population, statistical insight, comparison with other studies (these are examples of answers should you answer yes, you can describe in your own words as well).*

□Yes □No

_______________________________________________________________________________________________________________________________________________________________________________________________________________________________________________________________________________________________________________

12. Did the study attempt to increase sensitivity and/or specificity of AI/ML? (using tools, refine their inclusion/exclusion criteria, adjustment of cofounders, etc.…)

□Yes □No

________________________________________________________________________________________________________________________________________________________________________________________________________________________________________________________________________________________________________________

13. Did the use of AI/ML was more beneficial compared to Physicians only? *(If yes, can you mention in what aspect, (cost, length of stay, proper resistance patterns, faster results and therefore faster implementation, … etc.)*

□Yes □No

_______________________________________________________________________________________________________________________________________________________________________________________________________________________________________________________________________________________________________________________________________________________________________________________

14. When using AI, did the study state from where AI got their answers for diagnosis and treatment? (Guidelines, social media, journals. etc.), i*f yes, please mention percentage of each provided answer if mentioned in article.*

□Yes □No

_______________________________________________________________________________________________________________________________________________________________________________________________________________________________________________________________________________________________________________________________________________________________________________________

15. How did AI/ML/CDSS compare to Infectious Diseases in following guidelines and prescribing appropriate Antibiotics (if number or percentage are mentioned, please write them down*). Keep in mind, this will not be applicable for every articles.*

Did the paper compare the AI performance to that of providers?

|  | AI performance compared to providers |
| --- | --- |
| Considered as appropriate (or agreement) |  |
| Inappropriate (not agreement) |  |

Part 3. Unadjusted and adjusted associations

1. Raw numbers: Please fill raw data for the following tables if available. Only include measures of effect if they are listed in the manuscript. Please make sure that each sample is equivalent to one patient (if not specified, just assume it does).

- *Please note, there are different two by two tables, you will notice some articles will not fit into all of these tables (I have gone through the articles, and wrote done all the possibilities, should I have missed one, please free to adjust and add). Other articles, would not have the ability to fill the two-by-two table. Which is OK, and you can leave it blank.*

1. - two by two table concordance to guidelines

|  | | Usual care/Provider | | |
| --- | --- | --- | --- | --- |
|  |  | Yes | No | Total |
| CDSS/ML/AI +/-Prescriber | Yes |  |  |  |
|  | No |  |  |  |
|  | Total |  |  |  |

Sensitivity

Specificity

PPV

NPV

1. Two by two table, use of proper/optimal antibiotics

|  | | Usual care/Provider | | |
| --- | --- | --- | --- | --- |
|  |  | Yes | No | Total |
| CDSS/ML/AI +/-Prescriber | Yes |  |  |  |
|  | No |  |  |  |
|  | Total |  |  |  |

Sensitivity

Specificity

PPV

NPV

1. Two by two table, Effect of (CDSS+prescriber or prescriber alone) on length of antibiotics

|  | | Usual care/Provider | | |
| --- | --- | --- | --- | --- |
|  |  | Yes | No | Total |
| CDSS/ML/AI +/- Prescriber | Yes |  |  |  |
|  | No |  |  |  |
|  | Total |  |  |  |

Sensitivity

Specificity

PPV

NPV

1. Two by two table, early switch to oral antibiotics, using the aid of ML/AI in comparison to prescriber alone

|  | | Usual care/Provider | | |
| --- | --- | --- | --- | --- |
|  |  | Yes | No | Total |
| CDSS/ML/AI +/- Prescriber | Yes |  |  |  |
|  | No |  |  |  |
|  | Total |  |  |  |

Sensitivity

Specificity

PPV

NPV

*E. Should there anything you find significant for a two-by-two table, please add one.*

|  | | Usual care/Provider | | |
| --- | --- | --- | --- | --- |
|  |  | Yes | No | Total |
| CDSS/ML/AI +/-Prescriber | Yes |  |  |  |
|  | No |  |  |  |
|  | Total |  |  |  |

Sensitivity

Specificity

PPV

NPV

**Part 4.** Other references

Please look through the references. Are there other references that we should evaluate for the meta-analysis? If yes, please provide first author, journal and year _______________________________________________________________________________________________________________________________________________________________________________________________________________________________________________________________

Part 5: Quality Assessment Tool:

**Adapted Downs and Black Tool:**

1. Is the hypothesis/aim/objective of the study clearly described?

| yes | 1 |
| --- | --- |
| no | 0 |

2. Are the main outcomes to be measured clearly described in the Introduction or Methods section? If the main outcomes are first mentioned in the Results section, the question should be answered no.

| yes | 1 |
| --- | --- |
| no | 0 |

3. Are the characteristics of the participants include in the study clearly described?

In cohort and cross-sectional studies, inclusion and/or exclusion criteria should be given. In case-control studies, a case-definition and the sources for controls should be given.

| yes | 1 |
| --- | --- |
| no | 0 |

4. Are the interventions of interest clearly described?

AI/ML versus Usual care prescriber (where relevant) that are to be compared should be clearly described.

| yes | 1 |
| --- | --- |
| no | 0 |

5. Are the distributions of principal confounders in each group of subjects to be compared clearly described? A list of principal confounders is provided.

| yes | 2 |
| --- | --- |
| partially | 1 |
| no | 0 |

6. Are the main findings of the study clearly described?

Simple outcome data (including denominators and numerators) should be reported for all major findings so that the reader can check the major analyses and conclusions. (This question does not cover statistical tests which are considered below).

| yes | 1 |
| --- | --- |
| no | 0 |

7. Does the study provide estimates of the random variability in the data for the main outcomes?

In non-normally distributed data, the inter-quartile range of results should be reported. In normally distributed data the standard error, standard deviation or confidence intervals should be reported. If the distribution of the data is not described, it must be assumed that the estimates used were appropriate and the question should be answered yes.

| yes | 1 |
| --- | --- |
| no | 0 |

8. Have all important adverse events that may be a consequence of the intervention been reported?

This should be answered yes if the study demonstrates that there was a comprehensive attempt to measure adverse events. (A list of possible adverse events is provided).

| yes | 1 |
| --- | --- |
| no | 0 |

9. Have the characteristics of patients lost to follow-up been described?

This should be answered yes where there were no losses to follow-up or where losses to follow-up were so small that findings would be unaffected by their inclusion. This should be answered nowhere a study does not report the number of patients lost to follow-up.

| yes | 1 |
| --- | --- |
| no | 0 |

10. Have actual probability values been reported (e.g., 0.035 rather than <0.05) for the main outcomes except where the probability value is less than 0.001?

| yes | 1 |
| --- | --- |
| no | 0 |

**External validity:**

All the following criteria attempt to address the representativeness of the findings of the study and whether they may be generalized to the population from which the study subjects were derived.

11. Were the subjects asked to participate in the study representative of the entire population from which they were recruited?

The study must identify the source population for patients and describe how the patients were selected. Patients would be representative if they comprised the entire source population, an unselected sample of consecutive patients, or a random sample. Random sampling is only feasible where a list of all members of the relevant population exists. Where a study does not report the proportion of the source population from which the patients are derived, the question should be answered as unable to determine.

| yes | 1 |
| --- | --- |
| no | 0 |
| unable to determine | 0 |

12. Were those subjects who were prepared to participate representative of the entire population from which they were recruited?

The proportion of those asked who agreed should be stated. Validation that the sample was representative would include demonstrating that the distribution of the main confounding factors was the same in the study sample and the source population.

| yes | 1 |
| --- | --- |
| no | 0 |
| unable to determine | 0 |

 13. Were the staff, places, and facilities where the patients were treated, representative of the treatment the majority of patients receive?

For the question to be answered yes the study should demonstrate that the intervention was representative of that in use in the source population. The question should be answered no if, for example, the intervention was undertaken in a specialist centre unrepresentative of the hospitals most of the source population would attend.

| yes | 1 |
| --- | --- |
| no | 0 |
| unable to determine | 0 |

**Internal validity – bias**

14. Was an attempt made to blind study subjects to the intervention they have received ?

For studies where the patients would have no way of knowing which intervention they received, this should be answered yes.

| yes | 1 |
| --- | --- |
| no | 0 |
| unable to determine | 0 |

15. Was an attempt made to blind those measuring the main outcomes of the intervention?

| yes | 1 |
| --- | --- |
| no | 0 |
| unable to determine | 0 |

16. If any of the results of the study were based on “data dredging”, was this made clear?

Any analyses that had not been planned at the outset of the study should be clearly indicated. If no retrospective unplanned subgroup analyses were reported, then answer yes.

| yes | 1 |
| --- | --- |
| no | 0 |
| unable to determine | 0 |

17. In trials and cohort studies, do the analyses adjust for different lengths of follow-up of patients, or in case-control studies, is the time period between the intervention and outcome the same for cases and controls?

Where follow-up was the same for all study patients the answer should yes. If different lengths of follow-up were adjusted for by, for example, survival analysis the answer should be yes. Studies where differences in follow-up are ignored should be answered no.

| yes | 1 |
| --- | --- |
| no | 0 |
| unable to determine | 0 |

18. Were the statistical tests used to assess the main outcomes appropriate?

The statistical techniques used must be appropriate to the data. For example, non- parametric methods should be used for small sample sizes. Where little statistical analysis has been undertaken but where there is no evidence of bias, the question should be answered yes. If the distribution of the data (normal or not) is not described it must be assumed that the estimates used were appropriate and the question should be answered yes.

| yes | 1 |
| --- | --- |
| no | 0 |
| unable to determine | 0 |

19. Was compliance with the intervention/s reliable? Where there was noncompliance with the allocated treatment or where there was contamination of one group, the question should be answered no. For studies where the effect of any misclassification was likely to bias any association to the null, the question should be answered yes.

| yes | 1 |
| --- | --- |
| no | 0 |
| unable to determine | 0 |

20. Were the main outcome measures used accurate (valid and reliable)?

For studies where the outcome measures are clearly described, the question should be answered yes. For studies which refer to other work or that demonstrates the outcome measures are accurate, the question should be answered as yes.

| yes | 1 |
| --- | --- |
| no | 0 |
| unable to determine | 0 |

**Internal validity - confounding (selection bias)**

21. Were the patients in different intervention groups (trials and cohort studies) or were the cases and controls (case-control studies) recruited from the same population?

For example, patients for all comparison groups should be selected from the same hospital. The question should be answered unable to determine for cohort and case control studies where there is no information concerning the source of patients included in the study.

| yes | 1 |
| --- | --- |
| no | 0 |
| unable to determine | 0 |

22. Were study subjects in different intervention groups (trials and cohort studies) or were the cases and controls (case-control studies) recruited over the same period of time?

For a study which does not specify the time period over which patients were recruited, the question should be answered as unable to determine.

| yes | 1 |
| --- | --- |
| no | 0 |
| unable to determine | 0 |

23. Were study subjects randomized to intervention groups? Studies which state that subjects were randomized should be answered yes except where method of randomization would not ensure random allocation. For example, alternate allocation would score no because it is predictable.

| yes | 1 |
| --- | --- |
| no | 0 |
| unable to determine | 0 |

24. Was the randomized intervention assignment concealed from both patients and health care staff until recruitment was complete and irrevocable?

| yes | 1 |
| --- | --- |
| no | 0 |
| unable to determine | 0 |

25. Was there adequate adjustment for confounding in the analyses from which the main findings were drawn?

This question should be answered no for trials if: the main conclusions of the study were based on analyses of treatment rather than intention to treat; the distribution of known confounders in the different treatment groups was not described; or the distribution of known confounders differed between the treatment groups but was not taken into account in the analyses. In non-randomized studies if the effect of the main confounders was not investigated or con- founding was demonstrated but no adjustment was made in the final analyses the question should be answered as no.

| yes | 1 |
| --- | --- |
| no | 0 |
| unable to determine | 0 |

26. Were losses of patients to follow-up taken into account?

If the numbers of patients lost to follow-up are not reported, the question should be answered as unable to determine. If the proportion lost to follow-up was too small to affect the main findings, the question should be answered yes.

| yes | 1 |
| --- | --- |
| no | 0 |
| unable to determine | 0 |

**Power**

27. Did the study perform calculations to determine sufficient power to detect a clinically important difference?

Sample sizes have been calculated to detect a difference of x% and y%.

| Yes | 1 |
| --- | --- |
| No | 0 |

**Total score: ___**
